# Sic-transit—Another Bubble Burst

**Published:** 1885-08

**Authors:** 


### SIC TRANSIT—ANOTHER BUBBLE-BURST.

SO it appears that Ferran's boasted cholera prevention is a humbug! A writer in the *Chicago Tribune* says that he called on Mr. Ferran, and introduced himself as a pupil of Dr. Koch, but the Spaniard being suspicious, treated him with great discourtesy, snubbed him, insulted him even, by saying "you would steal my secret;" and the usher even opened the door, "intimating," the correspondent says, "that the interview was ended." Not to be baffled in any such way, the pseudo (or real, we don't know which) student got an apron and some other dress, and disguising himself as a tailor, staining his arms, face and neck brown, to complete the disguise, returned, and said he wanted to be vaccinated or inoculated. That was *business*; accordingly, the correspondent says, "He jabbed a sharp bistoury under my skin just above my left elbow, in a business-like manner. Then he took a bit of brownish unguental substance, and inserted it under the integument, covering the wound with a bit of court plaster, the whole proceeding reminding me of a rude way of vaccinating to protect from small-pox. The operation completed, the fellow told me that an eruption would appear at the seat of vaccination, and that violent catharsis ("induced cholera" he call it) would follow." He gave the student, also, three pills, and told him how to take them. The student says he hastened to his hotel, got there in twenty minutes, and locked himself in a room, goughed out the stuff that had been put in his arm, brought it under microscope, and submitted it to chemical analysis, and *mirabile dictu,* what do you suppose it was? Croton oil and elaterium, in vaseline! The most powerful drastic cathartics; the pills, too, being no more nor less than elaterium and croton oil, and to be taken at intervals of thirty-six hours for five days! The effect can be imagined; a condition very nearly resembling cholera, collapse, cramps and all. The correspondent says, "It is a grand piece of fraud," and closes as follows:

"I wonder at such a stroke of smartness in a Spaniard, and humbly hope he is not an American in disguise. The thing has the flavor of wooden nutmegs decidedly, and at any rate, the man is a genius who can gull thousands, and attract universal attention by taking the name of the cholera microbe in vain, and physicing people so deftly, that they have the redeeming symptoms of a disease that will not be trifled with in any such way."—*Journal A. M. A.*

This may be a *canard*, the work of an ingenious newspaper reporter for a sensation, but it sounds very like true. If this is true, the statistics given of the results of Ferran's inoculation in Alcia have a wonderful significance, if *they* are true. It looks almost incredible that the *effect of the imagination*, conjoined with a violent purging, induced by elaterium under deception, should apparently protect people from an attack of cholera. *Vive la bagatelle.*

